# Crystal structure of bis­(ammonium) bis­[penta­aqua­(di­methyl­formamide)­zinc(II)] deca­vanadate tetra­hydrate

**DOI:** 10.1107/S2056989022003449

**Published:** 2022-04-05

**Authors:** Arash Ebrahimi, Róbert Gyepes, Marek Bujdoš, Lukáš Krivosudský

**Affiliations:** a Comenius University in Bratislava, Faculty of Natural Sciences, Department of Inorganic Chemistry, Mlynská dolina, Ilkovičova 6, 842 15 Bratislava, Slovakia; b Charles University, Department of Inorganic Chemistry, Hlavova 2030, Prague, 128 00, Czech Republic; c Comenius University in Bratislava, Faculty of Natural Sciences, Institute of Laboratory Research on Geomaterials, Mlynská dolina, Ilkovičova 6, 842 15 Bratislava, Slovakia

**Keywords:** crystal structure, deca­vanadate, zinc complexes, polyoxometalate, hydrogen bonds

## Abstract

The crystalline product (NH_4_)_2_[Zn(C_3_H_7_NO)(H_2_O)_5_]_2_[V_10_O_28_]·4H_2_O was success­fully isolated from an H_2_O/DMF solvent combination by evaporation at ambient temperature. The salt crystallizes in the *P*2_1_/*n* space group. Imidazole, initially used in the synthesis but not present in the product, and DMF solvent appear to affect the synthesis and crystallization as structural-directing agents. In the title compound, the complex cation [Zn(H_2_O)_5_(DMF)]^2+^ acts as a counter-ion without being directly coordinated to the deca­vanadate anion. An extensive framework of hydrogen bonds integrates the whole architecture as evidenced by X-ray crystallography. The polyoxometalate [V_10_O_28_]^6–^ lies on a center of symmetry while the complex cation [Zn(H_2_O)_5_(DMF)]^2+^ links three adjacent anions through a set of 2 + 2 + 3 hydrogen bonds.

## Abstract 2

The title polyoxometalate salt, (NH_4_)_2_[Zn(DMF)(H_2_O)_5_]_2_[V_10_O_28_]·4H_2_O, comprising a cationic Zn^2+^ complex, belongs to a group of relatively rare deca­vanadates with complex counter-ions, previously including only eight crystal structures with zinc(II) components. In the crystal structure, the ions are linked by strong O—H⋯O hydrogen bonds between the coordinated water ligands and the deca­vanadate, with the O⋯O distances ranging from 2.660 (2) to 2.893 (2) Å.

## 1. Chemical context

Decavanadate anions, H_*x*_V_10_O_28_^(6–*x*)–^, are the major species in equilibrated aqueous vanadate solutions (Rehder, 2015 ▸; Gorzsás *et al.*, 2009 ▸) at vanadium(V) concentrations above 1 m*M* in the pH range of ≃2–6 (Schmidt *et al.*, 2001 ▸; Pettersson *et al.*, 1985 ▸), and are also stabilized in some organic solvents (Slebodnick & Pecoraro, 1998 ▸). There are altogether 54 compounds in the CSD (WebCSD, accessed January 2022; Groom *et al.*, 2016 ▸) that contain a deca­vanadate anion and a transition-metal complex cation, either coordinated or as a free counter-ion. Both groups are evenly abundant (27 structures). In our search for conditions under which the deca­vanadate acts as a ligand we focused on Zn^2+^ complexes that have already shown the ability to act as a counter-ion: (NH_4_)_2_[Zn (H_2_O)_6_]_2_[V_10_O_28_]·4H_2_O (Udomvech *et al.*, 2012 ▸), [Zn(H_2_O)_6_]_*n*_[{Na_2_(H_2_O)_6_(*μ_2_-*H_2_O)_4_Zn(H_2_O)_2_}V_10_O_28_]_*n*_·4*n*H_2_O (Yerra & Das, 2017 ▸), [Zn_3_(H*trz*)_6_(H_2_O)_6_][V_10_O_28_]·10H_2_O·H*trz* (Xu *et al.*, 2012 ▸), (C_4_H_14_N_2_)_2_]·[Zn(H_2_O)_6_][V_10_O_28_]·6H_2_O (Jin *et al.*, 2018 ▸), (NH_4_)_2_[Zn(H_2_O)_5_(NH_3_CH_2_CH_2_COO)]_2_[V_10_O_28_]·*n*H_2_O (Klištincová *et al.*, 2010 ▸); as well as being directly coordinated to deca­vanadate: {[Zn_2_(H_2_O)_14_[V_10_O_28_]}·H_2_ *ppz* (Wang *et al.*, 2008 ▸), {[Zn(*en*)_2_]_3_[V_10_O_28_]}·5H_2_O (Pang *et al.*, 2012 ▸), {[Zn(*im*)_2_(DMF)_2_]_2_[H_2_V_10_O_28_]·*im*·DMF (Xu *et al.*, 2012 ▸), {[Zn_3_(*trz*)_3_(H_2_O)_4_(DMF)]_2_[V_10_O_28_]·4H_2_O}_*n*_ (Xu *et al.*, 2012 ▸), [(CH_3_)_4_N]_2_[Zn(H_2_O)_5_]_2_[V_10_O_28_]}·5H_2_O (Huang *et al.*, 2021 ▸) and {[Zn(H_2_O)_6_][Zn_2_[V_10_O_28_](H_2_O)_10_]}·6H_2_O (Graia *et al.*, 2008 ▸) (*im* = imidazole, H*trz* = 1,2,4-triazole, DMF = *N*,*N*′-di­methyl­formamide, *en* = ethane-1,2-di­amine, *ppz* = piperazine). Employing zinc(II) centers as part of linker moieties for the construction of polyoxometalate-based metal organic frameworks (POMOFs) comes with an advantage over traditionally used rare metals regarding costs, and sometimes even efficiency. Important applications of POMOFs in materials chemistry include, for instance, photovoltaics (Luo *et al.*, 2012 ▸) and hydrogen evolution (Nohra *et al.*, 2011 ▸). Despite extensive experimental work with an inexpensive multicomponent system H_2_O/DMF/imidazole/Zn^2+^/V^5+^, we were not able to isolate from the various preparations any crystalline product other than (NH_4_)_2_[Zn(H_2_O)_5_(DMF)]_2_[V_10_O_28_]·4H_2_O (**1**). Its crystal structure is presented here. 
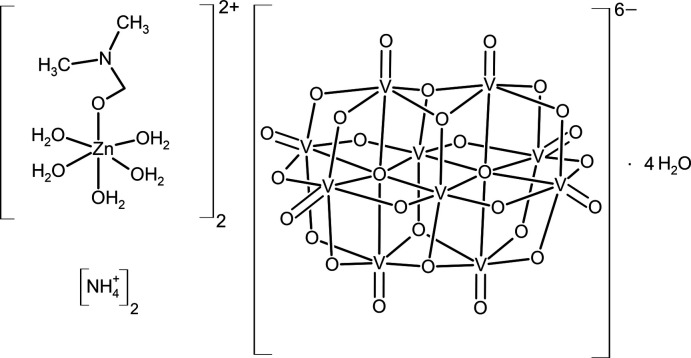


## 2. Structural commentary

**Figure 1:**
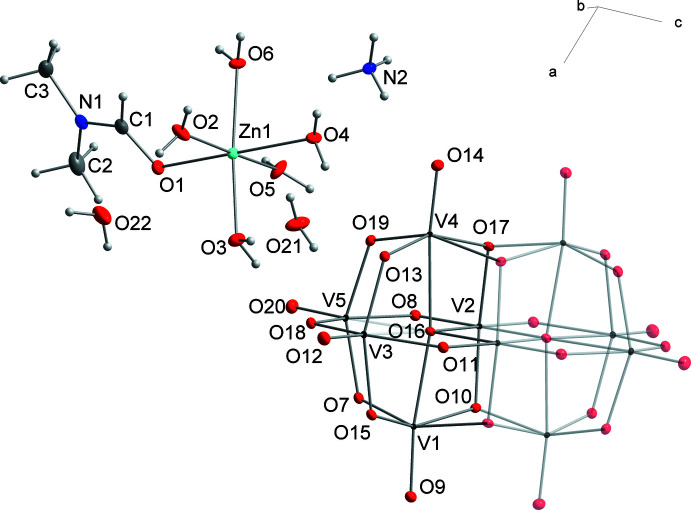
The mol­ecular structure of **1** showing 50% displacement ellipsoids illustrated with *DIAMOND* (Brandenburg & Putz, 2005 ▸). The half of the deca­vanadate anion that is not part of the asymmetric unit is displayed as faded.

Compound **1** crystallizes from a bicomponent solvent H_2_O/DMF at room temperature in the form of orange block-shaped crystals in monoclinic symmetry [*P*2_1_/*n*; β = 108.628 (1)°]. Although imidazole is not present in the crystal structure, neither as a free mol­ecule or cation nor as a ligand, its presence was necessary for crystallization to take place. In the absence of imidazole we observed the formation of oily solutions without crystalline product or the slow reduction of vanadium accompanied by a change in color of the solution from orange to greenish. The asymmetric unit of (NH_4_)_2_[Zn(H_2_O)_5_(DMF)]_2_[V_10_O_28_]·4H_2_O (Fig. 1 ▸) comprises one half of the [V_10_O_28_]^6–^ polyoxometalate, one [Zn(H_2_O)_5_(DMF)]^2+^ complex cation, one NH_4_^+^ and two mol­ecules of water of crystallization. The H atoms of the ammonium cation and water mol­ecules were found in the difference map and refined freely except for three water mol­ecules where restraints on the O—H distances were applied. The H atoms bound to the C atoms were placed in geometrically idealized positions and constrained to ride on their parent atoms. The Zn^2+^ center in [Zn(H_2_O)_5_(DMF)]^2+^ is coordinated by five aqua ligands with Zn—O bond lengths in the range 2.0482 (16)–2.1273 (16) Å and one *N*,*N*′-di­methyl­formamide ligand coordinated through the oxygen atom with a Zn—O bond length of 2.0926 (14) Å, forming an irregular octa­hedron. The deca­vanadate anion [V_10_O_28_]^6–^ is present in a fully deprotonated form, as further confirmed by elemental analysis and charge balance. It resides in a special position on the center of symmetry, as observed many times before (Rakovský & Krivosudský, 2014 ▸). The anion adopts *C*_i_ symmetry (idealized *D*_2*h*_) and is composed of ten edge-sharing heavily distorted octa­hedra. The terminal vanadium–oxygen bond lengths (V=O groups) are in the range 1.5929 (14)–1.6210 (14) Å, with an average value of 1.6083 Å. The bond lengths of the bridging μ–O atoms are in the range 1.6890 (13)–2.0696 (14) Å, with an average value of 1.853 Å. The bond lengths of the bridging *μ*_3_–O atoms with coord­ination numbers of three are in the range 1.8700 (14)–2.0208 (14) Å, with an average value of 1.9725 Å. Bond lengths of the hexa­coordinated oxygen atom trapped inside the deca­vanadate (O16) are in the range 2.1033 (13)–2.3337 (13) Å, with an average value of 2.2222 Å. All metrical parameters fall in their typical ranges.

## 3. Supra­molecular features

**Figure 2:**
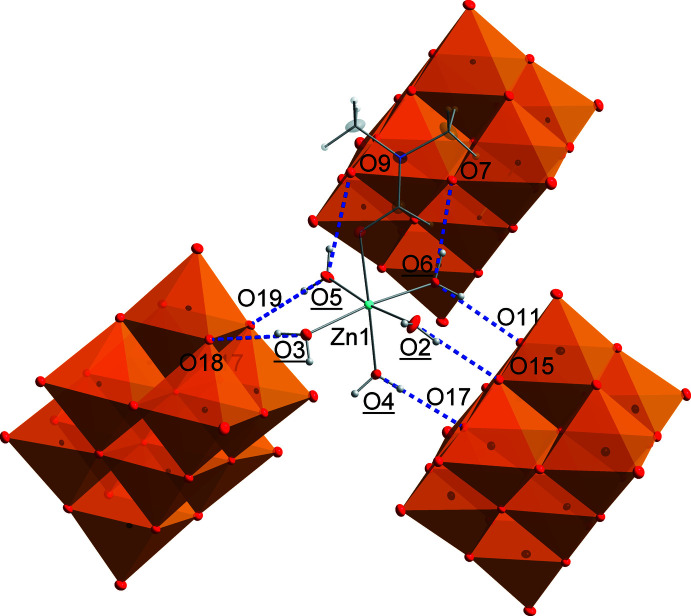
Relative positions of the three adjacent deca­vanadate anions (orange polyhedra) linked by a single [Zn(H_2_O)_5_(DMF)]^2+^ cation.

The supra­molecular structure of **1** is stabilized by a rich network of hydrogen bonds that involves all components of the compound. The strongest hydrogen bonds are formed by the complex cation (Fig. 2 ▸, Table 1 ▸), which serves as a linker for deca­vanadate anions in its vicinity. More specifically, [Zn(H_2_O)_5_(DMF)]^2+^ forms 2 + 2 + 3 hydrogen bonds through its aqua ligands (as donors) to three different [V_10_O_28_]^6−^ anions (as acceptors). The structural parameters of the hydrogen bonds are summarized in Table 1 ▸. Based on the *D*⋯*A* distances ranging from 2.659 (2) to 2.892 (2) Å and the angles *D*—H⋯*A* falling into the range 164 (3)–177 (3)°, the hydrogen bonds may be considered relatively strong examples.

## 4. Database survey

In a search of the Cambridge Structural Database (WebCSD, accessed January 2022; Groom *et al.*, 2016 ▸) for closely related deca­vanadates bearing mononuclear zinc(II) complex cations which are not coordinated to the deca­vanadate anion, six entries were found: (NH_4_)_2_[Zn(H_2_O)_6_]_2_[V_10_O_28_]·4H_2_O ICSD Entry: 422816 (Udomvech *et al.*, 2012 ▸), [Zn(H_2_O)_6_]_*n*_[{Na_2_(H_2_O)_6_(*μ-*H_2_O)_4_Zn(H_2_O)_2_}V_10_O_28_]_*n*_·4*n*H_2_O ICSD Entry: 427974 (Yerra & Das, 2017 ▸), (C_4_H_14_N_2_)_2_]·[Zn(H_2_O)_6_][V_10_O_28_]·6H_2_O YEYYEJ (Jin *et al.*, 2018 ▸), (NH_4_)_2_[Zn(H_2_O)_5_(NH_3_CH_2_CH_2_COO)]_2_[V_10_O_28_]·*n*H_2_O XABQIC (Klištincová *et al.*, 2010 ▸), [Zn(3-H*dpye*)(H_2_O)_5_]_2_[V_10_O_28_]·4H_2_O OXUYUD (Wang *et al.*, 2016 ▸), and [Zn(H_2_O)_6_][Na_3_(H_2_O)_14_][HV_10_O_28_]·4H_2_O SUDGUW (Amanchi & Das, 2018 ▸). The overall compositions (cations, deca­vanadate anion, water) are in all cases similar to that of the title compound.

## 5. Synthesis and crystallization

NH_4_VO_3_ (0.464 g, 4 mmol) was dissolved in 20 ml of water and stirred upon heating. After being cooled down to ambient temperature, deca­vanadate was prepared *in situ* by adjusting the pH to 4 with 2 *M* HCl until the color of the solution changed from bright yellow to orange. Under continuous stirring, imidazole (0.136 g, 2 mmol) was poured into the mixture and the pH was adjusted to 4 by adding 2 *M* HCl again. Finally, first ZnSO_4_·7H_2_O (0.287 g, 1 mmol) and secondly 20 mL of DMF were added to the clear solution. The mixture was filtered, and the clear orange filtrate was left to crystallize at RT. The orange crystals were isolated a few days later. The vanadium content was determined using an ICP MS Thermo Scientific iCap-Q; the zinc content was determined using an AAS Perkin-Elmer Model 1100. An infrared spectrum was recorded on a Nicolet FTIR 6700 spectrometer in Nujol mull. Analytical data for C_6_H_50_N_4_O_44_V_10_Zn_2_: theoretical V 33.5%, Zn 8.6%; found V 32.4%, Zn 8.40%. Characteristic bands in the FTIR spectrum (in cm^−1^): V_10_O_28_ 964, 951, 938, 805, 596; NH_4_^+^ 1416; DMF 1658, 1382, 1118.

## 6. Refinement

Crystal data, data collection and structure refinement details are summarized in Table 2 ▸. All non-hydrogen atoms were refined anisotropically. Hydrogen atoms were refined isotropically and those on carbon atoms were placed in geometrically idealized positions (C—H = 0.93 Å) and constrained to ride on their parent atoms with *U*_iso_(H) = 1.2 *U*_eq_(C). Hydrogen atoms of the water mol­ecules and the ammonium cation were found in the difference-Fourier map. For the two lattice water mol­ecules and one coordinated water, the O—H distances were restrained with DFIX while orientation and displacement parameters were refined freely. All other water hydrogen atoms and the ammonium cation hydrogen atoms were refined freely.

## Supplementary Material

Crystal structure: contains datablock(s) I. DOI: 10.1107/S2056989022003449/yz2017sup1.cif

Structure factors: contains datablock(s) I. DOI: 10.1107/S2056989022003449/yz2017Isup2.hkl

CCDC reference: 2156016

Additional supporting information:  crystallographic information; 3D view; checkCIF report

## Figures and Tables

**Table 1:** Hydrogen-bond geometry (Å, °)

*D*—H⋯*A*	*D*—H	H⋯*A*	*D*⋯*A*	*D*—H⋯*A*
O2—H2*O*⋯O15^i^	0.75 (3)	1.98 (3)	2.722 (2)	167 (3)
O3—H3*P*⋯O18	0.83 (3)	2.07 (3)	2.892 (2)	173 (3)
O4—H4*O*⋯O17^ii^	0.74 (3)	1.97 (3)	2.710 (2)	177 (3)
O5—H5*O*⋯O19	0.85 (3)	1.82 (3)	2.659 (2)	169 (3)
O5—H5*P*⋯O9^iii^	0.76 (3)	2.10 (3)	2.842 (2)	164 (3)
O6—H6*O*⋯O7^iii^	0.82 (3)	1.95 (3)	2.771 (2)	173 (3)
O6—H6*P*⋯O11^ii^	0.78 (2)	2.00 (2)	2.769 (2)	170 (3)

Symmetry codes: (i) 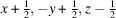 ; (ii) 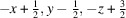 ; (iii) 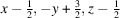 .

**Table 2:** Experimental details

Crystal data	
Chemical formula	(NH_4_)_2_[Zn(C_3_H_7_NO)(H_2_O)_5_]_2_[V_10_O_28_]·4H_2_O
*M*_r_	1522.64
Crystal system, space group	Monoclinic, *P*2_1_/*n*
Temperature (K)	120
*a*, *b*, *c* (Å)	15.5436 (6), 8.6538 (4), 16.7362 (7)
β (°)	108.628 (1)
*V* (Å^3^)	2133.27 (16)
*Z*	2
Radiation type	Mo *K*α
μ (mm^−1^)	3.31
Crystal size (mm)	0.49 × 0.23 × 0.10

Data collection	
Diffractometer	Nonius KappaCCD with Buker APEXII detector
Absorption correction	Multi-scan (*SADABS*; Krause *et al.*, 2015 ▸)
*T*_min_, *T*_max_	0.57, 0.73
No. of measured, independent and observed [*I* > 2σ(*I*)] reflections	29908, 4901, 4354
*R*_int_	0.033
(sin θ/λ)_max_ (Å^−1^)	0.650

Refinement	
*R*[*F* ^2^ > 2σ(*F* ^2^)], *wR*(*F* ^2^), *S*	0.023, 0.056, 1.06
No. of reflections	4901
No. of parameters	372
No. of restraints	6
H-atom treatment	H atoms treated by a mixture of independent and constrained refinement
Δρ_max_, Δρ_min_ (e Å^−3^)	0.36, −0.62

Computer programs: *Instrument Service* (Bruker, 2021 ▸), *SAINT* (Bruker, 2021 ▸), *SHELXT2018/2* (Sheldrick, 2015*a* ▸), *SHELXL2018/3* (Sheldrick, 2015*b* ▸) and *DIAMOND* (Brandenburg & Putz, 2005 ▸).

## supplementary crystallographic information

### Crystal data

**Table:**

(NH_4_)_2_[Zn(C_3_H_7_NO)(H_2_O)_5_]_2_[V_10_O_28_]·4H_2_O	*F*(000) = 1512
*M**_r_* = 1522.64	*D*_x_ = 2.370 Mg m^−^^3^
Monoclinic, *P*2_1_/*n*	Mo *K*α radiation, λ = 0.71073 Å
*a* = 15.5436 (6) Å	Cell parameters from 9949 reflections
*b* = 8.6538 (4) Å	θ = 2.6–27.5°
*c* = 16.7362 (7) Å	µ = 3.31 mm^−^^1^
β = 108.628 (1)°	*T* = 120 K
*V* = 2133.27 (16) Å^3^	Prism, orange
*Z* = 2	0.49 × 0.23 × 0.10 mm

### Data collection

**Table:**

Nonius KappaCCD with Buker APEXII detector diffractometer	4354 reflections with *I* > 2σ(*I*)
data from phi and ω scans	*R*_int_ = 0.033
Absorption correction: multi-scan (SADABS; Krause *et al.*, 2015)	θ_max_ = 27.5°, θ_min_ = 2.2°
*T*_min_ = 0.57, *T*_max_ = 0.73	*h* = −20→19
29908 measured reflections	*k* = −11→11
4901 independent reflections	*l* = −21→21

### Refinement

**Table:**

Refinement on *F*^2^	Primary atom site location: structure-invariant direct methods
Least-squares matrix: full	Hydrogen site location: mixed
*R*[*F*^2^ > 2σ(*F*^2^)] = 0.023	H atoms treated by a mixture of independent and constrained refinement
*wR*(*F*^2^) = 0.056	*w* = 1/[σ^2^(*F*_o_^2^) + (0.0284*P*)^2^ + 1.1852*P*] where *P* = (*F*_o_^2^ + 2*F*_c_^2^)/3
*S* = 1.06	(Δ/σ)_max_ = 0.002
4901 reflections	Δρ_max_ = 0.36 e Å^−^^3^
372 parameters	Δρ_min_ = −0.62 e Å^−^^3^
6 restraints	

### Special details

**Table:**

Geometry. All esds (except the esd in the dihedral angle between two l.s. planes) are estimated using the full covariance matrix. The cell esds are taken into account individually in the estimation of esds in distances, angles and torsion angles; correlations between esds in cell parameters are only used when they are defined by crystal symmetry. An approximate (isotropic) treatment of cell esds is used for estimating esds involving l.s. planes.

### Fractional atomic coordinates and isotropic or equivalent isotropic displacement parameters (Å^2^)

**Table:**

	*x*	*y*	*z*	*U*_iso_*/*U*_eq_	
Zn1	0.31800 (2)	0.46454 (3)	0.54363 (2)	0.00759 (6)	
O1	0.36049 (10)	0.62987 (16)	0.47301 (9)	0.0114 (3)	
O2	0.31034 (13)	0.30400 (19)	0.45168 (11)	0.0159 (3)	
H2O	0.276 (2)	0.240 (3)	0.4386 (19)	0.027 (9)*	
H2P	0.350 (2)	0.281 (4)	0.439 (2)	0.043 (11)*	
O3	0.44977 (11)	0.3899 (2)	0.60794 (10)	0.0125 (3)	
H3O	0.4493 (17)	0.310 (3)	0.6204 (16)	0.012 (7)*	
H3P	0.478 (2)	0.433 (3)	0.653 (2)	0.027 (8)*	
O4	0.27068 (12)	0.31855 (17)	0.62168 (10)	0.0107 (3)	
H4O	0.2351 (18)	0.260 (3)	0.6034 (16)	0.012 (7)*	
H4P	0.311 (2)	0.274 (3)	0.6540 (19)	0.026 (8)*	
O5	0.32040 (12)	0.64584 (19)	0.62565 (10)	0.0147 (3)	
H5O	0.338 (2)	0.648 (4)	0.679 (2)	0.037 (9)*	
H5P	0.309 (2)	0.728 (4)	0.609 (2)	0.041 (10)*	
O6	0.17863 (11)	0.51996 (18)	0.48529 (10)	0.0107 (3)	
H6O	0.1613 (18)	0.600 (3)	0.4584 (16)	0.025 (8)*	
H6P	0.1389 (16)	0.463 (3)	0.4648 (17)	0.018 (7)*	
C1	0.31089 (15)	0.6545 (2)	0.39870 (13)	0.0127 (4)	
H1	0.267624	0.577353	0.372375	0.015*	
N1	0.31404 (12)	0.7783 (2)	0.35441 (11)	0.0126 (4)	
C2	0.37449 (17)	0.9071 (3)	0.38953 (16)	0.0207 (5)	
H2A	0.339767	0.991473	0.403582	0.031*	
H2B	0.402328	0.943400	0.348019	0.031*	
H2C	0.422078	0.873260	0.440631	0.031*	
C3	0.24580 (16)	0.8028 (3)	0.27197 (15)	0.0230 (5)	
H3A	0.208105	0.710072	0.255621	0.035*	
H3B	0.276127	0.823842	0.229986	0.035*	
H3C	0.207451	0.891060	0.275052	0.035*	
V1	0.66396 (2)	0.54542 (4)	0.97966 (2)	0.00519 (8)	
V2	0.48442 (2)	0.68753 (4)	0.99591 (2)	0.00468 (8)	
V3	0.54965 (2)	0.33633 (4)	0.83349 (2)	0.00615 (8)	
V4	0.37025 (2)	0.46974 (4)	0.84932 (2)	0.00550 (8)	
V5	0.51936 (2)	0.68687 (4)	0.82367 (2)	0.00627 (8)	
O7	0.63516 (9)	0.70057 (15)	0.90117 (8)	0.0068 (3)	
O8	0.48640 (9)	0.80713 (15)	0.91677 (8)	0.0066 (3)	
O9	0.76998 (9)	0.57672 (15)	1.03142 (9)	0.0079 (3)	
O10	0.60927 (9)	0.67399 (15)	1.05199 (8)	0.0057 (3)	
O11	0.45500 (9)	0.79640 (15)	1.06789 (8)	0.0066 (3)	
O12	0.57404 (10)	0.20530 (16)	0.77742 (9)	0.0102 (3)	
O13	0.42410 (9)	0.32537 (15)	0.79962 (8)	0.0067 (3)	
O14	0.26409 (10)	0.43520 (16)	0.80457 (9)	0.0096 (3)	
O15	0.66637 (9)	0.39324 (15)	0.90763 (8)	0.0068 (3)	
O16	0.51531 (9)	0.50621 (14)	0.92593 (8)	0.0055 (3)	
O17	0.36261 (9)	0.60759 (15)	0.94154 (8)	0.0061 (3)	
O18	0.54495 (10)	0.51674 (15)	0.77235 (9)	0.0072 (3)	
O19	0.39504 (9)	0.63135 (15)	0.79227 (8)	0.0070 (3)	
O20	0.51633 (10)	0.82531 (16)	0.75840 (9)	0.0107 (3)	
N2	0.13802 (14)	0.5436 (2)	0.65462 (12)	0.0091 (4)	
H2R	0.0918 (19)	0.476 (3)	0.6319 (17)	0.017 (7)*	
H2S	0.176 (2)	0.502 (3)	0.691 (2)	0.024 (8)*	
H2T	0.1165 (18)	0.620 (3)	0.6764 (16)	0.019 (7)*	
H2Q	0.165 (2)	0.572 (3)	0.618 (2)	0.031 (8)*	
O21	0.41424 (12)	0.08923 (19)	0.64987 (12)	0.0191 (4)	
H21A	0.446 (2)	0.032 (3)	0.6837 (18)	0.036 (9)*	
H21B	0.3783 (19)	0.042 (3)	0.6175 (18)	0.037 (10)*	
O22	0.46542 (13)	0.2601 (2)	0.41289 (12)	0.0260 (4)	
H22A	0.472 (2)	0.250 (4)	0.3693 (16)	0.040 (10)*	
H22B	0.5092 (18)	0.296 (4)	0.4460 (18)	0.039 (10)*	

### Atomic displacement parameters (Å^2^)

**Table:**

	*U*^11^	*U*^22^	*U*^33^	*U*^12^	*U*^13^	*U*^23^
Zn1	0.00799 (12)	0.00689 (11)	0.00765 (11)	−0.00058 (9)	0.00214 (9)	−0.00008 (9)
O1	0.0120 (8)	0.0121 (7)	0.0107 (7)	−0.0031 (6)	0.0046 (6)	0.0005 (6)
O2	0.0122 (9)	0.0163 (8)	0.0222 (9)	−0.0065 (7)	0.0099 (7)	−0.0108 (7)
O3	0.0122 (8)	0.0103 (8)	0.0122 (8)	0.0005 (7)	0.0001 (6)	0.0005 (7)
O4	0.0087 (8)	0.0085 (7)	0.0129 (8)	−0.0020 (7)	0.0006 (7)	0.0017 (6)
O5	0.0246 (9)	0.0090 (8)	0.0068 (8)	0.0014 (7)	−0.0002 (7)	−0.0004 (6)
O6	0.0079 (8)	0.0077 (7)	0.0133 (8)	−0.0006 (6)	−0.0009 (6)	0.0015 (6)
C1	0.0122 (11)	0.0143 (10)	0.0135 (10)	0.0001 (9)	0.0069 (9)	−0.0010 (8)
N1	0.0106 (9)	0.0150 (9)	0.0142 (9)	0.0021 (7)	0.0069 (8)	0.0037 (7)
C2	0.0288 (14)	0.0143 (11)	0.0244 (12)	−0.0034 (10)	0.0162 (11)	0.0011 (9)
C3	0.0163 (12)	0.0374 (14)	0.0168 (12)	0.0070 (11)	0.0073 (10)	0.0110 (11)
V1	0.00446 (17)	0.00613 (15)	0.00523 (15)	0.00007 (12)	0.00188 (13)	−0.00022 (12)
V2	0.00508 (17)	0.00396 (15)	0.00497 (15)	0.00072 (12)	0.00156 (13)	0.00013 (12)
V3	0.00678 (17)	0.00668 (16)	0.00561 (16)	0.00051 (13)	0.00282 (13)	−0.00102 (12)
V4	0.00511 (17)	0.00675 (15)	0.00438 (15)	0.00021 (12)	0.00115 (13)	−0.00009 (12)
V5	0.00768 (18)	0.00618 (16)	0.00514 (16)	0.00000 (13)	0.00230 (13)	0.00097 (12)
O7	0.0071 (7)	0.0072 (6)	0.0068 (6)	−0.0008 (5)	0.0032 (6)	−0.0003 (5)
O8	0.0068 (7)	0.0058 (6)	0.0074 (7)	0.0003 (5)	0.0024 (6)	0.0003 (5)
O9	0.0070 (7)	0.0088 (6)	0.0081 (7)	0.0001 (6)	0.0027 (6)	0.0000 (5)
O10	0.0057 (7)	0.0055 (6)	0.0056 (6)	−0.0004 (5)	0.0011 (5)	−0.0006 (5)
O11	0.0068 (7)	0.0061 (6)	0.0072 (7)	0.0007 (5)	0.0027 (6)	0.0000 (5)
O12	0.0104 (8)	0.0113 (7)	0.0097 (7)	0.0000 (6)	0.0045 (6)	−0.0033 (6)
O13	0.0066 (7)	0.0076 (6)	0.0061 (6)	0.0000 (5)	0.0023 (6)	−0.0003 (5)
O14	0.0078 (7)	0.0121 (7)	0.0081 (7)	−0.0005 (6)	0.0014 (6)	0.0002 (6)
O15	0.0060 (7)	0.0077 (6)	0.0075 (7)	0.0001 (5)	0.0033 (6)	0.0000 (5)
O16	0.0045 (7)	0.0057 (6)	0.0063 (6)	0.0001 (5)	0.0019 (6)	0.0004 (5)
O17	0.0045 (7)	0.0062 (6)	0.0075 (6)	0.0009 (5)	0.0019 (5)	0.0002 (5)
O18	0.0071 (7)	0.0084 (6)	0.0064 (6)	−0.0006 (5)	0.0029 (6)	−0.0002 (5)
O19	0.0074 (7)	0.0079 (6)	0.0055 (6)	0.0012 (5)	0.0017 (6)	0.0014 (5)
O20	0.0117 (8)	0.0109 (7)	0.0095 (7)	−0.0005 (6)	0.0033 (6)	0.0018 (6)
N2	0.0090 (9)	0.0078 (8)	0.0095 (9)	0.0005 (8)	0.0014 (8)	0.0011 (7)
O21	0.0162 (9)	0.0113 (8)	0.0217 (9)	−0.0007 (7)	−0.0056 (8)	−0.0002 (7)
O22	0.0184 (10)	0.0405 (11)	0.0223 (10)	−0.0112 (8)	0.0110 (9)	−0.0143 (9)

### Geometric parameters (Å, º)

**Table:**

Zn1—O2	2.0482 (16)	V2—O11	1.7029 (14)
Zn1—O5	2.0772 (16)	V2—O10	1.8700 (14)
Zn1—O3	2.0886 (16)	V2—O17	1.9470 (14)
Zn1—O1	2.0926 (14)	V2—O16	2.1033 (13)
Zn1—O4	2.1113 (16)	V2—O16^i^	2.1256 (13)
Zn1—O6	2.1273 (16)	V2—V3^i^	3.0726 (5)
O1—C1	1.255 (3)	V2—V5	3.0993 (5)
O2—H2O	0.75 (3)	V3—O12	1.5929 (14)
O2—H2P	0.74 (3)	V3—O13	1.8525 (14)
O3—H3O	0.73 (3)	V3—O18	1.8552 (14)
O3—H3P	0.83 (3)	V3—O15	1.9067 (14)
O4—H4O	0.74 (3)	V3—O11^i^	2.0310 (14)
O4—H4P	0.79 (3)	V3—O16	2.3168 (14)
O5—H5O	0.84 (3)	V3—V5	3.0662 (5)
O5—H5P	0.76 (3)	V3—V4	3.1066 (5)
O6—H6O	0.82 (2)	V4—O14	1.6074 (15)
O6—H6P	0.78 (2)	V4—O19	1.8031 (14)
C1—N1	1.313 (3)	V4—O13	1.8434 (14)
C1—H1	0.9500	V4—O17	1.9841 (14)
N1—C2	1.455 (3)	V4—O10^i^	2.0101 (14)
N1—C3	1.463 (3)	V4—O16	2.2317 (14)
C2—H2A	0.9800	V4—V5	3.1181 (5)
C2—H2B	0.9800	V5—O20	1.6118 (14)
C2—H2C	0.9800	V5—O18	1.8115 (14)
C3—H3A	0.9800	V5—O7	1.8559 (14)
C3—H3B	0.9800	V5—O19	1.8954 (14)
C3—H3C	0.9800	V5—O8	2.0696 (14)
V1—O9	1.6210 (14)	V5—O16	2.3337 (13)
V1—O15	1.7939 (14)	N2—H2R	0.91 (3)
V1—O7	1.8312 (14)	N2—H2S	0.79 (3)
V1—O17^i^	2.0027 (14)	N2—H2T	0.87 (3)
V1—O10	2.0208 (14)	N2—H2Q	0.88 (3)
V1—O16	2.2215 (14)	O21—H21A	0.79 (2)
V1—V4^i^	3.0765 (5)	O21—H21B	0.76 (2)
V1—V5	3.1011 (5)	O22—H22A	0.78 (2)
V1—V3	3.1047 (5)	O22—H22B	0.79 (2)
V2—O8	1.6890 (13)		
			
O2—Zn1—O5	173.36 (7)	O11^i^—V3—V2^i^	31.29 (4)
O2—Zn1—O3	89.40 (7)	O16—V3—V2^i^	43.72 (3)
O5—Zn1—O3	94.90 (7)	V5—V3—V2^i^	92.705 (12)
O2—Zn1—O1	89.57 (7)	O12—V3—V1	134.03 (5)
O5—Zn1—O1	85.10 (6)	O13—V3—V1	123.55 (4)
O3—Zn1—O1	93.90 (6)	O18—V3—V1	81.71 (4)
O2—Zn1—O4	96.37 (7)	O15—V3—V1	31.85 (4)
O5—Zn1—O4	88.82 (6)	O11^i^—V3—V1	81.33 (4)
O3—Zn1—O4	88.52 (7)	O16—V3—V1	45.57 (3)
O1—Zn1—O4	173.62 (6)	V5—V3—V1	60.334 (11)
O2—Zn1—O6	90.11 (7)	V2^i^—V3—V1	62.101 (11)
O5—Zn1—O6	86.20 (6)	O12—V3—V4	134.74 (5)
O3—Zn1—O6	173.47 (6)	O13—V3—V4	32.71 (4)
O1—Zn1—O6	92.61 (6)	O18—V3—V4	81.72 (4)
O4—Zn1—O6	85.06 (6)	O15—V3—V4	122.81 (4)
C1—O1—Zn1	118.11 (14)	O11^i^—V3—V4	82.82 (4)
Zn1—O2—H2O	126 (2)	O16—V3—V4	45.79 (3)
Zn1—O2—H2P	123 (3)	V5—V3—V4	60.676 (11)
H2O—O2—H2P	107 (3)	V2^i^—V3—V4	61.307 (10)
Zn1—O3—H3O	110 (2)	V1—V3—V4	91.121 (12)
Zn1—O3—H3P	118 (2)	O14—V4—O19	104.99 (7)
H3O—O3—H3P	103 (3)	O14—V4—O13	102.14 (7)
Zn1—O4—H4O	121 (2)	O19—V4—O13	94.71 (6)
Zn1—O4—H4P	111 (2)	O14—V4—O17	99.59 (6)
H4O—O4—H4P	107 (3)	O19—V4—O17	91.24 (6)
Zn1—O5—H5O	130 (2)	O13—V4—O17	155.11 (6)
Zn1—O5—H5P	121 (2)	O14—V4—O10^i^	97.90 (7)
H5O—O5—H5P	108 (3)	O19—V4—O10^i^	155.58 (6)
Zn1—O6—H6O	123.2 (19)	O13—V4—O10^i^	88.61 (6)
Zn1—O6—H6P	127.5 (19)	O17—V4—O10^i^	76.46 (5)
H6O—O6—H6P	102 (3)	O14—V4—O16	172.94 (6)
O1—C1—N1	125.2 (2)	O19—V4—O16	81.20 (6)
O1—C1—H1	117.4	O13—V4—O16	80.45 (6)
N1—C1—H1	117.4	O17—V4—O16	76.62 (5)
C1—N1—C2	122.21 (19)	O10^i^—V4—O16	75.50 (5)
C1—N1—C3	120.29 (19)	O14—V4—V1^i^	88.21 (5)
C2—N1—C3	116.75 (19)	O19—V4—V1^i^	130.95 (4)
N1—C2—H2A	109.5	O13—V4—V1^i^	128.99 (4)
N1—C2—H2B	109.5	O17—V4—V1^i^	39.72 (4)
H2A—C2—H2B	109.5	O10^i^—V4—V1^i^	40.38 (4)
N1—C2—H2C	109.5	O16—V4—V1^i^	85.11 (4)
H2A—C2—H2C	109.5	O14—V4—V3	134.99 (5)
H2B—C2—H2C	109.5	O19—V4—V3	83.90 (4)
N1—C3—H3A	109.5	O13—V4—V3	32.89 (4)
N1—C3—H3B	109.5	O17—V4—V3	124.63 (4)
H3A—C3—H3B	109.5	O10^i^—V4—V3	85.94 (4)
N1—C3—H3C	109.5	O16—V4—V3	48.08 (3)
H3A—C3—H3C	109.5	V1^i^—V4—V3	119.349 (14)
H3B—C3—H3C	109.5	O14—V4—V5	138.20 (5)
O9—V1—O15	104.24 (7)	O19—V4—V5	33.46 (4)
O9—V1—O7	103.57 (7)	O13—V4—V5	83.20 (4)
O15—V1—O7	96.26 (6)	O17—V4—V5	88.66 (4)
O9—V1—O17^i^	98.45 (6)	O10^i^—V4—V5	123.81 (4)
O15—V1—O17^i^	90.58 (6)	O16—V4—V5	48.31 (3)
O7—V1—O17^i^	154.50 (6)	V1^i^—V4—V5	120.716 (13)
O9—V1—O10	97.95 (6)	V3—V4—V5	59.021 (11)
O15—V1—O10	155.49 (6)	O20—V5—O18	104.25 (7)
O7—V1—O10	88.44 (6)	O20—V5—O7	103.77 (7)
O17^i^—V1—O10	75.81 (5)	O18—V5—O7	94.15 (6)
O9—V1—O16	171.99 (6)	O20—V5—O19	101.19 (7)
O15—V1—O16	81.84 (6)	O18—V5—O19	91.23 (6)
O7—V1—O16	80.63 (6)	O7—V5—O19	152.28 (6)
O17^i^—V1—O16	76.05 (5)	O20—V5—O8	100.13 (6)
O10—V1—O16	75.19 (5)	O18—V5—O8	155.54 (6)
O9—V1—V4^i^	87.61 (5)	O7—V5—O8	81.94 (6)
O15—V1—V4^i^	129.85 (4)	O19—V5—O8	81.98 (6)
O7—V1—V4^i^	128.56 (4)	O20—V5—O16	173.31 (6)
O17^i^—V1—V4^i^	39.28 (4)	O18—V5—O16	82.22 (5)
O10—V1—V4^i^	40.12 (4)	O7—V5—O16	77.15 (5)
O16—V1—V4^i^	84.47 (4)	O19—V5—O16	76.68 (5)
O9—V1—V5	136.35 (5)	O8—V5—O16	73.35 (5)
O15—V1—V5	83.63 (5)	O20—V5—V3	137.94 (5)
O7—V1—V5	32.99 (4)	O18—V5—V3	33.71 (4)
O17^i^—V1—V5	124.67 (4)	O7—V5—V3	85.86 (4)
O10—V1—V5	87.51 (4)	O19—V5—V3	83.66 (4)
O16—V1—V5	48.63 (3)	O8—V5—V3	121.86 (4)
V4^i^—V1—V5	120.389 (14)	O16—V5—V3	48.51 (3)
O9—V1—V3	138.31 (5)	O20—V5—V2	130.78 (5)
O15—V1—V3	34.12 (4)	O18—V5—V2	124.94 (5)
O7—V1—V3	85.11 (4)	O7—V5—V2	76.63 (4)
O17^i^—V1—V3	86.96 (4)	O19—V5—V2	78.01 (4)
O10—V1—V3	123.27 (4)	O8—V5—V2	30.65 (4)
O16—V1—V3	48.13 (4)	O16—V5—V2	42.72 (3)
V4^i^—V1—V3	118.865 (14)	V3—V5—V2	91.233 (12)
V5—V1—V3	59.218 (11)	O20—V5—V1	136.08 (5)
O8—V2—O11	106.98 (7)	O18—V5—V1	82.44 (5)
O8—V2—O10	98.98 (6)	O7—V5—V1	32.50 (4)
O11—V2—O10	98.61 (6)	O19—V5—V1	122.27 (4)
O8—V2—O17	96.31 (6)	O8—V5—V1	81.48 (4)
O11—V2—O17	94.96 (6)	O16—V5—V1	45.59 (3)
O10—V2—O17	155.59 (6)	V3—V5—V1	60.448 (11)
O8—V2—O16	87.47 (6)	V2—V5—V1	60.851 (11)
O11—V2—O16	165.32 (6)	O20—V5—V4	132.78 (5)
O10—V2—O16	81.25 (6)	O18—V5—V4	82.01 (5)
O17—V2—O16	80.53 (5)	O7—V5—V4	122.67 (4)
O8—V2—O16^i^	165.68 (6)	O19—V5—V4	31.63 (4)
O11—V2—O16^i^	87.09 (6)	O8—V5—V4	79.96 (4)
O10—V2—O16^i^	81.02 (6)	O16—V5—V4	45.57 (3)
O17—V2—O16^i^	79.50 (5)	V3—V5—V4	60.303 (11)
O16—V2—O16^i^	78.36 (6)	V2—V5—V4	60.938 (10)
O8—V2—V3^i^	145.25 (5)	V1—V5—V4	90.971 (12)
O11—V2—V3^i^	38.27 (5)	V1—O7—V5	114.51 (7)
O10—V2—V3^i^	89.35 (4)	V2—O8—V5	110.69 (7)
O17—V2—V3^i^	88.85 (4)	V2—O10—V4^i^	108.53 (6)
O16—V2—V3^i^	127.24 (4)	V2—O10—V1	107.55 (6)
O16^i^—V2—V3^i^	48.87 (4)	V4^i^—O10—V1	99.50 (6)
O8—V2—V5	38.66 (5)	V2—O11—V3^i^	110.45 (7)
O11—V2—V5	145.64 (5)	V4—O13—V3	114.40 (7)
O10—V2—V5	90.27 (4)	V1—O15—V3	114.03 (7)
O17—V2—V5	89.87 (4)	V2—O16—V2^i^	101.64 (6)
O16—V2—V5	48.82 (4)	V2—O16—V1	93.07 (5)
O16^i^—V2—V5	127.18 (4)	V2^i^—O16—V1	94.24 (5)
V3^i^—V2—V5	176.038 (14)	V2—O16—V4	93.27 (5)
O12—V3—O13	102.07 (7)	V2^i^—O16—V4	92.58 (5)
O12—V3—O18	104.42 (7)	V1—O16—V4	169.57 (7)
O13—V3—O18	91.28 (6)	V2—O16—V3	170.95 (7)
O12—V3—O15	102.19 (7)	V2^i^—O16—V3	87.41 (5)
O13—V3—O15	154.51 (6)	V1—O16—V3	86.30 (5)
O18—V3—O15	90.18 (6)	V4—O16—V3	86.13 (5)
O12—V3—O11^i^	98.81 (6)	V2—O16—V5	88.46 (5)
O13—V3—O11^i^	84.97 (6)	V2^i^—O16—V5	169.89 (7)
O18—V3—O11^i^	156.74 (6)	V1—O16—V5	85.77 (5)
O15—V3—O11^i^	83.72 (6)	V4—O16—V5	86.12 (5)
O12—V3—O16	173.76 (6)	V3—O16—V5	82.50 (4)
O13—V3—O16	77.99 (5)	V2—O17—V4	106.64 (6)
O18—V3—O16	81.80 (5)	V2—O17—V1^i^	107.55 (6)
O15—V3—O16	77.04 (5)	V4—O17—V1^i^	101.01 (6)
O11^i^—V3—O16	74.96 (5)	V5—O18—V3	113.48 (7)
O12—V3—V5	137.23 (5)	V4—O19—V5	114.91 (7)
O13—V3—V5	84.58 (4)	H2R—N2—H2S	110 (3)
O18—V3—V5	32.81 (4)	H2R—N2—H2T	108 (2)
O15—V3—V5	82.95 (4)	H2S—N2—H2T	109 (3)
O11^i^—V3—V5	123.94 (4)	H2R—N2—H2Q	112 (3)
O16—V3—V5	48.99 (3)	H2S—N2—H2Q	104 (3)
O12—V3—V2^i^	130.07 (5)	H2T—N2—H2Q	114 (2)
O13—V3—V2^i^	78.67 (4)	H21A—O21—H21B	109 (3)
O18—V3—V2^i^	125.52 (4)	H22A—O22—H22B	111 (3)
O15—V3—V2^i^	79.82 (4)		
			
Zn1—O1—C1—N1	160.84 (16)	V1^i^—V4—O13—V3	−84.80 (8)
O1—C1—N1—C2	−3.0 (3)	V5—V4—O13—V3	39.62 (6)
O1—C1—N1—C3	−172.8 (2)	O12—V3—O13—V4	−177.51 (8)
O9—V1—O7—V5	174.37 (7)	O18—V3—O13—V4	−72.47 (8)
O15—V1—O7—V5	68.06 (8)	O15—V3—O13—V4	20.64 (18)
O17^i^—V1—O7—V5	−36.62 (17)	O11^i^—V3—O13—V4	84.54 (7)
O10—V1—O7—V5	−87.82 (7)	O16—V3—O13—V4	8.87 (7)
O16—V1—O7—V5	−12.58 (7)	V5—V3—O13—V4	−40.31 (6)
V4^i^—V1—O7—V5	−87.71 (8)	V2^i^—V3—O13—V4	53.56 (6)
V3—V1—O7—V5	35.75 (6)	V1—V3—O13—V4	8.43 (9)
O20—V5—O7—V1	−174.70 (8)	O9—V1—O15—V3	−177.41 (7)
O18—V5—O7—V1	−68.93 (8)	O7—V1—O15—V3	−71.66 (8)
O19—V5—O7—V1	31.69 (17)	O17^i^—V1—O15—V3	83.73 (7)
O8—V5—O7—V1	86.78 (7)	O10—V1—O15—V3	28.39 (18)
O16—V5—O7—V1	12.11 (7)	O16—V1—O15—V3	7.90 (7)
V3—V5—O7—V1	−36.23 (6)	V4^i^—V1—O15—V3	83.63 (8)
V2—V5—O7—V1	56.03 (6)	V5—V1—O15—V3	−41.12 (6)
V4—V5—O7—V1	14.23 (9)	O20—V5—O18—V3	−178.51 (8)
O11—V2—O8—V5	−179.02 (6)	O7—V5—O18—V3	76.15 (8)
O10—V2—O8—V5	79.05 (7)	O19—V5—O18—V3	−76.64 (8)
O17—V2—O8—V5	−81.86 (7)	O8—V5—O18—V3	−3.5 (2)
O16—V2—O8—V5	−1.67 (7)	O16—V5—O18—V3	−0.26 (7)
O16^i^—V2—O8—V5	−9.8 (3)	V2—V5—O18—V3	−0.44 (10)
V3^i^—V2—O8—V5	−178.97 (3)	V1—V5—O18—V3	45.77 (6)
O8—V2—O10—V4^i^	−178.45 (6)	V4—V5—O18—V3	−46.29 (6)
O11—V2—O10—V4^i^	72.71 (7)	O12—V3—O18—V5	−179.26 (8)
O17—V2—O10—V4^i^	−50.33 (16)	O13—V3—O18—V5	77.94 (8)
O16—V2—O10—V4^i^	−92.44 (6)	O15—V3—O18—V5	−76.62 (8)
O16^i^—V2—O10—V4^i^	−12.94 (6)	O11^i^—V3—O18—V5	−2.3 (2)
V3^i^—V2—O10—V4^i^	35.44 (5)	O16—V3—O18—V5	0.27 (7)
V5—V2—O10—V4^i^	−140.62 (5)	V2^i^—V3—O18—V5	0.97 (10)
O8—V2—O10—V1	−71.68 (7)	V1—V3—O18—V5	−45.80 (6)
O11—V2—O10—V1	179.48 (6)	V4—V3—O18—V5	46.56 (6)
O17—V2—O10—V1	56.44 (16)	O14—V4—O19—V5	−174.03 (7)
O16—V2—O10—V1	14.33 (6)	O13—V4—O19—V5	−70.12 (8)
O16^i^—V2—O10—V1	93.83 (6)	O17—V4—O19—V5	85.69 (8)
V3^i^—V2—O10—V1	142.21 (5)	O10^i^—V4—O19—V5	26.93 (18)
V5—V2—O10—V1	−33.84 (5)	O16—V4—O19—V5	9.44 (7)
O8—V2—O11—V3^i^	179.95 (7)	V1^i^—V4—O19—V5	85.04 (8)
O10—V2—O11—V3^i^	−77.85 (7)	V3—V4—O19—V5	−39.01 (6)
O17—V2—O11—V3^i^	81.80 (7)	O20—V5—O19—V4	177.33 (8)
O16—V2—O11—V3^i^	10.5 (3)	O18—V5—O19—V4	72.55 (8)
O16^i^—V2—O11—V3^i^	2.61 (7)	O7—V5—O19—V4	−28.78 (17)
V5—V2—O11—V3^i^	178.87 (3)	O8—V5—O19—V4	−83.86 (8)
O14—V4—O13—V3	177.55 (8)	O16—V5—O19—V4	−9.16 (7)
O19—V4—O13—V3	71.10 (8)	V3—V5—O19—V4	39.64 (6)
O17—V4—O13—V3	−32.15 (18)	V2—V5—O19—V4	−52.98 (6)
O10^i^—V4—O13—V3	−84.67 (8)	V1—V5—O19—V4	−9.28 (9)
O16—V4—O13—V3	−9.14 (7)		

Symmetry code: (i) −*x*+1, −*y*+1, −*z*+2.

### Hydrogen-bond geometry (Å, º)

**Table:**

*D*—H···*A*	*D*—H	H···*A*	*D*···*A*	*D*—H···*A*
O2—H2*O*···O15^ii^	0.75 (3)	1.98 (3)	2.722 (2)	167 (3)
O3—H3*P*···O18	0.83 (3)	2.07 (3)	2.892 (2)	173 (3)
O4—H4*O*···O17^iii^	0.74 (3)	1.97 (3)	2.710 (2)	177 (3)
O5—H5*O*···O19	0.85 (3)	1.82 (3)	2.659 (2)	169 (3)
O5—H5*P*···O9^iv^	0.76 (3)	2.10 (3)	2.842 (2)	164 (3)
O6—H6*O*···O7^iv^	0.82 (3)	1.95 (3)	2.771 (2)	173 (3)
O6—H6*P*···O11^iii^	0.78 (2)	2.00 (2)	2.769 (2)	170 (3)

Symmetry codes: (ii) *x*+1/2, −*y*+1/2, *z*−1/2; (iii) −*x*+1/2, *y*−1/2, −*z*+3/2; (iv) *x*−1/2, −*y*+3/2, *z*−1/2.
